# The Impact of Living Arrangements and Intergenerational Support on the Health Status of Older People in China

**DOI:** 10.1093/geroni/igab046.2916

**Published:** 2021-12-17

**Authors:** Yazhen Yang, Maria Evandrou, Athina Vlachantoni

**Affiliations:** University of Southampton, Southampton, England, United Kingdom

## Abstract

Research to-date has examined the impact of intergenerational support in terms of isolated types of support, or at one point in time, failing to provide strong evidence of the complex effect of support on older persons’ wellbeing. Using the Harmonised China Health and Retirement Longitudinal Study (2011, 2013 and 2015), this paper investigates the impact of older people’s living arrangements and intergenerational support provision/ receipt on their physical and psychological wellbeing, focusing on rural/ urban differences. The results show that receiving economic support from one’s adult children was a stronger predictor for higher life satisfaction among older rural residents compared to those in urban areas, while grandchild care provision was an important determinant for poor life satisfaction only for older urban residents. Receiving informal care from one’s adult children was associated with a poor (I)ADL functional status and with depressive symptoms among older rural people. Meanwhile, having weekly in-person and distant contact reduced the risk of depression among older people in both rural and urban areas. The paper shows that it is important to improve the level of public economic transfers and public social care towards vulnerable older people in rural areas, and more emphasis should be placed on improving the psychological well-being of urban older residents, such as with the early diagnosis of depression.

